# Comparative effectiveness of physical activity interventions and anti-hypertensive pharmacological interventions in reducing blood pressure in people with hypertension: protocol for a systematic review and network meta-analysis

**DOI:** 10.1186/s13643-018-0791-9

**Published:** 2018-08-21

**Authors:** C. Noone, C. P. Dwyer, J. Murphy, J. Newell, G. J. Molloy

**Affiliations:** 1 School of Psychology, National University of Ireland, Galway, Newcastle Road, Galway, H91 TK33 Ireland; 2 School of Mathematics, Statistics & Applied Mathematics, National University of Ireland, Galway, Newcastle Road, Galway, H91 TK33 Ireland

**Keywords:** Hypertension, Blood pressure, Physical activity, Anti-hypertensives, Network meta-analysis

## Abstract

**Background:**

The prevalence of hypertension is a major public health challenge. Despite it being highly preventable, hypertension is responsible for a significant proportion of global morbidity and mortality. Common methods for controlling hypertension include prescribing anti-hypertensive medication, a pharmacological approach, and increasing physical activity, a behavioural approach. In general, little is known about the comparative effectiveness of pharmacological and behavioural approaches for reducing blood pressure in hypertension. A previous network meta-analysis suggested that physical activity interventions may be just as effective as many anti-hypertensive medications in preventing mortality; however, this analysis did not provide the comparative effectiveness of these disparate modes of intervention on blood pressure reduction. The primary objective of this study is to use network meta-analysis to compare the relative effectiveness, for blood pressure reduction, of different approaches to increasing physical activity and different first-line anti-hypertensive therapies in people with hypertension.

**Methods:**

A systematic review will be conducted to identify studies involving randomised controlled trials which compare different types of physical activity interventions and first-line anti-hypertensive therapy interventions to each other or to other comparators (e.g. placebo, usual care) where blood pressure reduction is the primary outcome. We will search the Cochrane Library, MEDLINE and PsycInfo. For studies which meet our inclusion criteria, two reviewers will extract data independently and assess the quality of the literature using the Cochrane Risk of Bias Tool. Network meta-analyses will be conducted to generate estimates of comparative effectiveness of each intervention class and rankings of their effectiveness, in terms of reduction of both systolic and diastolic blood pressure.

**Discussion:**

This study will provide evidence regarding the comparability of two common first-line treatment options for people with hypertension. It will also describe the extent to which there is direct evidence regarding the comparative effectiveness of increasing physical activity and initiating anti-hypertensive therapy.

**Systematic review registration:**

PROSPERO CRD42017070579

**Electronic supplementary material:**

The online version of this article (10.1186/s13643-018-0791-9) contains supplementary material, which is available to authorized users.

## Background

Hypertension, defined as systolic blood pressure of 140 mmHg or greater or diastolic blood pressure of 90 mmHg or greater [1], affects 1 billion people worldwide and is a key risk factor for heart attack, stroke and renal failure [2, 3]. Though hypertension is one of the most preventable contributors to disease and death [4–6], it remains a major public health problem, responsible for approximately 7.5–9.4 million deaths worldwide annually [7, 8]. Thus, further emphasis on approaches aimed at treating and preventing hypertension, through modifying behavioural risk factors, is essential to controlling this growing epidemic [9]. Behaviour plays a critical role in the maintenance of an individual’s health, as well as in the primary and secondary prevention of many chronic diseases worldwide [10].

An extensive body of research suggests physical activity may have beneficial effects on blood pressure in people living with hypertension [11]. Hypertension management through the intervention of medication has also been shown to have substantive benefits in reducing blood pressure and primary prevention of morbidity and mortality from cardiovascular diseases [12, 13]. Despite both physical activity intervention and anti-hypertensive pharmacological intervention being recommended as first-line treatments for hypertension, few studies have directly compared them.

There is a need for such comparisons as demonstrated by a recent research prioritisation effort involving healthcare providers, caregivers and people living with hypertension. The top priority identified in this study was the following question: ‘What healthy lifestyle habits or combination of habits can reduce or eliminate the need for anti-hypertensive agents?’ [14]. People living with hypertension often take more than one anti-hypertensive medications and have been shown to hold negative beliefs regarding their medication. These beliefs and the side-effects produced by anti-hypertensive medication interact to contribute to generally low medication adherence among people living with hypertension [15, 16]. Physical activity appears to be an attractive alternative to anti-hypertensive medication as a first-line treatment due to its comparatively fewer and less severe side-effects. However, it should be noted that physical activity is not without its adherence challenges.

Valid indirect comparisons between physical activity and anti-hypertensive have become possible due to the advent of advanced statistical techniques, such as network meta-analysis (NMA) [17]. For example, Naci and Ioannidis employed NMA to assess the comparative effectiveness of several drugs, including some anti-hypertensive medications, and physical activity across a range of conditions. However, hypertension was not one of the conditions studied. This analysis showed that physical activity interventions likely has a similar effect on mortality as many anti-hypertensive drug interventions for patients with stroke, heart failure, coronary heart disease and prediabetes [17]. This study will employ NMA to focus on the comparative effectiveness of physical activity interventions and anti-hypertensive drug interventions for lowering blood pressure in people living with hypertension.

NMA is a quantitative methodology that provides a global estimate of comparative treatment effectiveness, through combining both direct and indirect evidence [18]. For example, specific to the context of the current research, NMA will allow for comparison of the efficacy of behavioural (i.e. physical activity) and biomedical (pharmacological treatment) intervention strategies [17], that would otherwise not have been previously compared in head-to-head trials, on a common health outcome (i.e. blood pressure), provided they share common comparator(s); for example, a control or placebo condition. Indirect comparison between studies through NMA has become a critical component of evidence synthesis and decision-making in healthcare [19], perhaps because opportunities for such comparison provide an attractive proposition to clinicians, given their concern with identifying the ‘single best’ treatment available [18].

The primary aim of the current research is to examine the comparative effectiveness on blood pressure reduction in people living hypertension of physical activity interventions versus first-line anti-hypertensive medications (i.e. calcium channel blockers, angiotensin receptor blockers, thiazide diuretics and ACE inhibitors) as both are considered viable first-line treatments according to the recommendations of the National Institute for Health and Care Excellence Guidelines (NICE; [26]).

## Methods

We adhere to the Preferred Reporting Items for Systematic Review and Meta-Analysis Protocols (PRISMA-P) in this protocol [20]. Similarly, the results of this study will be reported in line with the PRISMA extension for network meta-analyses [21]. We will also apply the International Society for Pharmacoeconomics and Outcomes Research (ISPOR) Indirect Treatment Comparison/Network Meta-Analysis Study Questionnaire to Assess Relevance and Credibility to Inform Health Care Decision-Making to our study to aid the interpretation of clinicians and other healthcare decision-makers [22]. This study is registered in the PROSPERO database, and any amendments to this protocol will be indicated there.

### Eligibility criteria

#### Population

The population of interest is adults, between the ages of 18 and 75, with a diagnosis of hypertension or with poorly controlled blood pressure (i.e. SBP ≥ 140 mmHg; DBP ≥ 90 mmHg). Samples where more than 30% of the sample is diabetic will be excluded (as in [23]). In addition, samples with hypertension due to pregnancy, following stroke or solely due to an acute situation (e.g. hypertensive crisis, whitecoat hypertension) will be excluded.

#### Interventions

The systematic review will focus on interventions which are intended to reduce blood pressure in people with hypertension using either physical activity or first-line anti-hypertensive therapy. The first-line anti-hypertensive therapy options are derived from the National Institute for Health and Care Excellence guidelines for the diagnosis and management of hypertension in adults [24]. These include calcium channel blockers, angiotensin receptor blockers, thiazide diuretics and ACE inhibitors. The term ‘physical activity intervention’ captures the broad range of interventions focused at increasing the expenditure of energy to above resting levels [25, 26]. Interventions focused on increasing physical activity in people with hypertension have included aerobic exercise training, resistance training, combined aerobic and resistance training [27], high intensity interval training, accumulated exercise interventions [25], yoga training [28] and walking-based interventions [29].

#### Comparators

There are a number of types of comparator conditions which will be eligible for inclusion in the network of evidence. The types of control conditions may include no treatment, pharmacological placebo, treatment as usual or active behavioural control groups. Furthermore, different types of physical activity interventions and anti-hypertensive therapies may also be directly compared.

#### Outcomes

The primary outcome is the mean difference in blood pressure, both systolic and diastolic, between the beginning of the intervention and follow-up. This will be assessed for studies that report the outcome in terms of a continuous difference score measured in mmHg.

#### Study designs

The systematic review will only include randomised controlled trials. These trials must have a sample size of at least 20 participants per condition and must involve interventions delivered for a minimum of 4 weeks. To reduce heterogeneity, crossover trials will be excluded. Studies making within-class comparisons only (e.g. a calcium channel blocker versus an alternative calcium channel blocker) will also be excluded. Finally, studies must be reported in English.

### Information sources and search strategy

The search strategy was developed in consultation with an information specialist in the university library and also reviewed by an independent information specialist. This strategy targeted databases relevant to health and behavioural science including PsycINFO (Ovid interface, from 1806 onwards), The Cochrane Library (Wiley interface, current issue) and MEDLINE (Ovid interface, from 1946 onwards). It draws search terms from existing meta-analyses of anti-hypertensives (e.g. [13, 30]) and physical activity interventions (e.g. [17, 25–32]). Separate searches will be performed for physical activity intervention trials and anti-hypertensive trials (see Additional file 1).

### Data collection and analysis

#### Study selection

Detailed citations for the studies identified through the application of the search strategy will be exported to EndNote [33]. Automatic and manual de-duplication will be carried out in EndNote. This library of citations will then be exported and uploaded to Covidence [34], an online system for screening and extracting data from papers for the purposes of a systematic review. Two independent reviewers will use Covidence to examine the titles and abstracts of all papers gathered during the literature search. Both reviewers will screen a random 10% of the papers in duplicate. This will establish consistency and allow for any discrepancies in approach to be resolved through discussion. We will assess consistency using the kappa statistic. The rest of the papers will be screened at this stage by the two review authors independently. Studies which are passed through this initial screening stage will be examined in more detail using the full text of the study report. Study authors will be contacted in cases where information is not completely enough to make a screening decision (e.g. conference abstracts, brief reports, lack of required detail in reporting). The reasons for exclusion will be documented in Covidence. A PRISMA flowchart will be exported from Covidence to document the screening process [35]. The data will be extracted in Covidence, which implements a customisable data extraction form for both reviewers.

#### Data extraction

Data extraction will be performed on all studies which are passed through the full-text screening stage by two independent reviewers. Again, we will assess consistency using the kappa statistic. This will be conducted in Covidence using a custom data extraction form. This data extraction form will be piloted on a random sample of ten included studies in order to ensure at least 80% agreement. The form may be altered in order to improve agreement and ease of use. Any disagreement will resolved by a third reviewer. In cases where data from a single study population is reported in multiple studies, the study which reports the fullest blood pressure reduction data (i.e. with the largest sample size) will be the source for data extraction. Other related studies will be used solely for supplementary information. For multi-arm trials, we will include all arms following the guidance of Dias et al. [36].

The data to be extracted includes authorship list, year, journal of publication, countries of study, funding source, intervention type, comparator(s), total study duration, setting, diagnostic criteria, group sample sizes, mean SBP pre-intervention, mean DBP pre-intervention, mean SBP at follow-up, mean DBP at follow-up, study inclusion criteria, age distribution, gender distribution, ethnicity distribution, patient comorbidity history, past/present medication use and key conclusions of the study authors. For all studies (including those with multiple follow-ups), follow-up points will be classified as short-term (≤ 90 days), medium-term (91–364 days), or long-term (> 364 days) follow-up. The data will be exported to a .csv file from Covidence.

#### Classification of arms

Classification of arms will be carried out at the data extraction stage. The arms of each trial will be classified according to the type of physical activity intervention, type of anti-hypertensive drug class administered or type of control condition employed, where applicable. The classes of anti-hypertensive drug included in this study are those used as first-line treatment, as indicated by the NICE guidelines [24], and include calcium channel blockers, angiotensin receptor blockers, thiazide diuretics and ACE inhibitors. The physical activity interventions will be classified according to the different types reviewed by Ghadieh and Saab [27] in their review of physical activity interventions for the treatment of hypertension. These classifications will include dynamic resistance training, isometric resistance training, aerobic training and combination training, which may include combinations of the any of the previously listed types of physical activity. See Table 1 for operational definitions of these classifications. If any disagreements regarding classification cannot be resolved by a third reviewer, the project advisory group, which comprises academics with a wide range of relevant expertise, will be consulted and the classification will be determined by consensus. Control conditions will be classified as active or passive. Passive control conditions are those where no intervention is applied, while active control conditions involve some intervention (e.g. placebo, usual care).

**Table 1:** Classifications of physical activity interventions

Physical activity intervention class	Definition	Examples
Aerobic training	Regular and purposeful movement of joints and large muscle groups	Walking, jogging, running, cycling, swimming
Dynamic resistance training	Exertion of effort against an opposing force alongside by purposeful movement of joints and large muscle groups	Weight-lifting, circuit training with resistance-training equipment
Isometric resistance training	Contraction of muscles with no change in the length of the involved muscle groups and without joint movement for a sustained period of time	Plank, wall sit
Combination training	Any combination of the above	

A network diagram will be generated to visualise the evidence available for analysis, both in terms of possible pairwise comparisons and the volume of evidence underlying each of these comparisons [37]. The reference node will contain active control conditions. Different combinations of interventions will be included as separate nodes.

#### Risk of bias

For studies which are passed through the full-text screening stage, risk of bias will be assessed independently by two reviewers using the standard Cochrane risk of bias tool in Covidence which assesses sequence generation and allocation concealment, blinding of participants and personnel, blinding of outcome assessors and incompleteness of the outcome data and whether reporting appears to be selective [38].

#### Summary measures

Summary measures will be reported for systolic blood pressure (SBP) and diastolic blood pressure (DBP). For studies where continuous data is employed, the summary measures produced will be mean differences between treatment arms (with 95% credible intervals). The mean difference will be obtained either by subtracting the BP at the follow-up from the BP at the baseline, though some papers explicitly state the difference. Any studies for which we cannot obtain two out of the three of baseline, change and follow-up will be excluded. In addition, we will exclude any study that does not give either the standard deviation of the change or the standard deviation at the follow-up (or allows us to compute this through confidence intervals or standard errors). We expect that these decisions will reduce bias while maintaining an informative evidence network. The data will also be summarised using treatment rankings and a surface under the cumulative ranking curve to aid the interpretation of the comparative effectiveness of all intervention types included in the network.

#### Assessment of transitivity

A table of trial characteristics which may act as effect modifiers will be compiled from the data collected (as specified above) to aid in the assessment of the assumption of transitivity [39, 40]. Potential effect modifiers include total study duration, setting, age distribution, gender distribution, ethnicity distribution, patient comorbidity history and past/present medication use.

### Data synthesis

The main objective of this data synthesis is to compare the effectiveness of different interventions focused on increasing physical activity or taking anti-hypertensive medication. NMA is useful for achieving this objective because it allows indirect estimates to be computed where little direct evidence exists. Few studies have directly compared physical activity interventions to anti-hypertensive therapy. Studies will be pooled according to the trial arm classifications noted above. If quantitatively pooling the study results is not possible, the findings of the systematic review will be described narratively. All quantitative analyses will be carried out using Stata 14 [41] and WinBUGS [42].

#### Pairwise meta-analysis

Where head-to-head data is available, exploratory pairwise meta-analyses will be conducted. These will be run using a random-effects model. The individual and pooled effect sizes will be visualised using Forrest plots. These will be conducted for both SBP and DBP outcomes. Funnel plots and Egger’s test will be employed to examine publication bias and the effects of small studies [43].

#### Network meta-analysis

If the assumption of transitivity is deemed to be met, random-effects NMAs will be conducted within a Bayesian framework using vague priors. Again, these will be conducted for both SBP and DBP outcomes. These analyses will be carried out in line with the framework set out by Dias et al. [36]. Estimates of the pairwise comparison of each intervention in the network will be presented in tables in the final manuscript, as will rankings demonstrating the probability of each intervention producing the best outcome. These rankings will be presented with mean ranks, 95% credible intervals and the surface under the cumulative ranking curve. Convergence will be assessed by checking if the Gelman-Rubin statistic is less than 1.1 [44, 45].

#### Assessment of inconsistency, heterogeneity and quality of the evidence

Statistical heterogeneity will be assessed for each pairwise meta-analysis using the *I*^2^ and *Tau*^2^ statistics in line with the Cochrane guidelines. Since the included studies are likely to consist of a mixture of two-arm and multi-arm studies, it is necessary to consider design inconsistency as well as loop inconsistency. This will be achieved by applying a design-by-treatment interaction model. If inconsistency is indicated in the network, any closed loops within the network will be assessed [46].

The quality of the evidence used in this study will be assessed using the GRADE four-step approach for rating the quality of treatment effect estimates from NMA [47].

#### Additional analyses

Exploratory analyses will be carried out, where there is a sufficient amount of information available to do so. These analyses will focus on the covariates the age distribution, gender distribution and ethnicity distribution. Network meta-regressions will be conducted to individually examine the influence of these covariates on effect size estimates. Finally, sensitivity analyses will be conducted to assess the influence of the use of specific treatments in the network rather than classes and trial quality.

## Discussion

### Contribution to literature

Hypertension remains a major public health problem, responsible for approximately 7.5–9.4 million deaths worldwide annually, even though it is one of the most preventable contributors to disease and death. Research indicates that behaviour plays a critical role in an individual’s health management and that common daily behaviours, such as medication adherence and physical activity, have beneficial effects on hypertension. However, there is a dearth of research directly comparing the effects of treatment through medication and physical activity, which has made it difficult to assess which intervention yields the best outcome for hypertensive patients. To date, there is no clear indication as to which intervention is the most effective.

However, in recent times, medical literature has witnessed a rapid increase in the possibility to combine evidence coming from a set of treatment comparisons [18], namely through network meta-analysis (NMA). In the current research, NMA will allow for comparison of the efficacy of physical activity and pharmacological treatment intervention strategies [17], which would otherwise not have been previously compared in head-to-head trials on blood pressure. Results from the NMA will address and fill gaps in the extant literature through making synthesised, evidence-based suggestions regarding the efficacy and superiority of the interventions and will allow for recommendation regarding future health resource allocation.

Furthermore, the NMA will quantitatively compare different forms of physical activity and a variety of anti-hypertensive medications in order to identify the most efficacious. Specifically, physical activities and medications will be ranked in order to determine the most (and least) effective interventions, which will provide evidence-based justification for choosing certain anti-hypertensive medications and/or physical activities over others.

### Limitations

Though the NMA methodology has become a critical component of evidence synthesis and decision-making in healthcare, there are potential drawbacks that may require consideration, such as the observational nature of indirect comparisons, assumptions that underlie the model and issues with inconsistency [48]. However, such issues may be overcome through appropriate and conservative employment of NMA, in which case opportunities to foster new research and medical decisions in a particular direction outweigh the potential drawbacks of using this methodology [49].

### Implications of the review

To our knowledge, no previous review has conducted a NMA comparing the effects of pharmacological treatment and physical activity on reducing blood pressure in hypertension. The current review will provide a clear direction for future research in the area of behaviour change to increase physical activity for people with hypertension.

## Additional files

Additional file 1: Search strategy. (PDF 4733 kb)

## Abbreviations

ACE: Angiotensin-converting enzyme
DBP: Diastolic blood pressure
DIC: Deviance information criterion
NICE: National Institute for Clinical Excellence
NMA: Network meta-analysis
PRISMA: Preferred reporting items for systematic reviews and meta-analyses
SBP: Systolic blood pressure
UK: United Kingdom
